# Account of Diseases in an Eastern District of London, from the 20th of February, to the 20th of March, 1807

**Published:** 1807-04

**Authors:** 


*Account of Diseases in an Eastern District of London, from the 20th of February, to the 20th of March,* 1807.

| | |
|---|---|
| **Acute Diseases.** | |
| Pneumonia | 5 |
| Peripneumonia Notha | 7 |
| Catarrhus | 8 |
| Cynanche Tonsillaris | 2 |
| Enteritis | 1 |
| Rheumatismus Acutus | 3 |
| **Chronic Diseases.** | |
| Tussis | 18 |
| Tussis cum Dyspnœa | 20 |
| Phthisis Pulmonalis | 3 |
| Asthma | 1 |
| Hæmoptysis | 2 |
| Dyspepsia | 4 |
| Vomitus | 3 |
| Gastrodynia | 5 |
| Ascites | 2 |
| Anasarca | 4 |
| Syphilis | 2 |
| Menorrhagia | 5 |
| Amenorrhœa | 7 |
| Fluor Albus | 5 |
| Cephalalgia | 5 |
| Vertigo | 3 |
| Paralysis | 2 |
| Palpitatio | 3 |
| Rheumatismus Chronicus | 30 |
| **Puerperal Diseases.** | |
| Ephemera | 3 |
| Menorrhagia Lochialis | 3 |
| Rhagas Papillæ | 4 |
| **Infantile Diseases.** | |
| Erysipelas Infantilis | 1 |
| Aphthæ | 4 |
| Ophthalmia | 2 |
| Tabes Mesenterica | 3 |

The diseases which have occurred during the last few weeks are very similar to those which were taken notice of in the last Report. Coughs, catarrhs, difficulty of respiration, and all the train of pneumonic affections, have made their appearance. These symptoms have been considerably aggravated by the severity of the weather. The long continuance of North and North-Easterly winds, has proved a severe trial to patients labouring under the diseases referred to; and some of those, who from the uncommon mildness of the weather, during the preceding weeks, weeks, were flattered with the hope of getting through the winter with tolerable comfort, have suffered from disappointment; and with some of the aged and infirm the disease has terminated fatally. In one of the cases of Peripneumony, a considerable degree of sickness and vomiting was connected with it. This symptom, when it occurs, may arise from the sympathy which subsists between the lungs and the stomach, and in some instances, though unpleasant to bear, may be productive of some beneficial consequences. During this state of the stomach, expectoration is generally more free, and thus a favourable termination of the disease is procured.

